# Purulent Pericardial Effusion: An Elusive Diagnosis

**DOI:** 10.7759/cureus.113141

**Published:** 2026-07-22

**Authors:** Shaker Al-Nabulsi, Dania Hammadi, Ahmed Abozenah, Mohammed Abozenah, Julia Ladna

**Affiliations:** 1 Medicine, Royal College of Surgeons in Ireland - Bahrain, Muharraq, BHR; 2 Internal Medicine, Newgiza University, Cairo, EGY; 3 Cardiovascular Medicine, University of Massachusetts Medical School - Baystate Medical Center, Springfield, USA; 4 Cardiovascular Medicine, Baystate Medical Center, Springfield, USA

**Keywords:** cardiac tamponade, fungal pericarditis, pericardiocentesis, polymicrobial infection, purulent pericardial effusion, tuberculous pericarditis

## Abstract

Purulent pericardial effusion (PPE) is a life-threatening infection of the pericardial space. While rare in the modern antimicrobial era, its rapidly progressive course, diagnostic challenges, and lack of management guidance make it a source of significant morbidity and mortality. Gram-positive bacteria are the most common causative organisms; however, polymicrobial and fungal infections are being increasingly recognized. Rapid diagnosis requires a high index of suspicion supported by clinical findings, pericardial fluid analysis, and imaging studies. Prompt drainage of the infected pericardial fluid along with early antimicrobial therapy remain the cornerstones of management, although gaps on optimal treatment duration, need for surgical intervention, and follow-up persist. This review aims to bridge some of these gaps by examining the current literature on purulent pericardial effusion, highlighting the epidemiology, microbiology, clinical manifestations, diagnostic challenges, management strategies, and both short-term and long-term outcomes of this deadly condition.

## Introduction and background

Pericardial effusion, the accumulation of fluid within the pericardial sac, is a common cardiac condition that affects up to 6.5% of the general adult population worldwide [1]. It can present variably, from asymptomatic incidental discoveries to life-threatening complications such as cardiac tamponade [2]. Usually occurring in the context of pericarditis, numerous conditions can predispose to it. Of them, idiopathic, infectious, inflammatory, traumatic, and neoplastic etiologies are most common [3]. Frankly purulent pericardial effusion (PPE) is much less common, however.

A narrative review of the literature was conducted on published case reports, case series, and review articles on purulent pericarditis and PPE. Special emphasis was placed on literature discussing reports of polymicrobial, fungal, and tuberculous infections, as well as on the currently described management guidelines. This review summarizes the epidemiology, microbiology, clinical presentation, diagnostic approach, management strategies, and outcomes of PPE with an emphasis on diagnostic cues that may improve early recognition and treatment.

Literature search strategy

A narrative review was conducted by searching PubMed through December 2025, with no start date restriction. Various combinations of search terms were used, including "purulent pericarditis," "bacterial pericarditis," "fungal pericarditis," "pericardial empyema," "tuberculous pericarditis," "pericardiocentesis," "cardiac tamponade," "pericardial drainage," "pericardiectomy," and "polymicrobial pericarditis." Other articles were identified through hand-searching the reference lists of relevant publications.

Articles were selected if they included clinically relevant information on adult purulent pericardial effusion, comprising microbiology, epidemiology, diagnostic findings, clinical presentations, management, and outcomes. Pediatric and non-English reports were excluded. Given the rarity of PPE and the predominance of case reports and small case series, most with different populations, organisms, management, and outcomes, a meta-analysis was not feasible. Given the narrative design of this review, a formal risk-of-bias assessment of individual studies was not performed. The methodological limitations of the included studies are addressed in the limitations section. As such, findings were summarized narratively, and conclusions were drawn with appropriate caution due to the limitations of the available evidence.

## Review

Epidemiology and risk factors

Purulent pericardial effusion (PPE) accounts for less than 1% of all pericardial effusion cases [4]. It too has a variable course, but unlike non-purulent pericardial effusion, it is generally associated with significant morbidity and mortality, with mortality rates up to 40% despite adequate treatment and up to 100% if left untreated [5]. This condition can impact children as young as five months old [6] and the very elderly. However, the average age affected by this condition has increased from young adolescents to adults aged 49 over the last 60 years [7]. PPE is most common in immunocompromised individuals but can occur in the immunocompetent in the context of active malignancy or invasive cardiothoracic procedures [7]. A wide range of causative organisms has been identified, with bacterial pathogens being the most prevalent, although fungal organisms are increasingly being identified [8,9]. Given its rapidly progressive course, it is imperative to maintain a high index of suspicion, as prompt recognition and early intervention are crucial to improve outcomes and reduce mortality [10].

Microbiology and routes of infection

Numerous classification schemes for PPE have been proposed, although no unified system exists. The majority of cases are secondary, attributable to an underlying infection, although primary infections are still diagnosed with some frequency [11]. Bacterial pathogens remain the most prevalent causative organisms, with gram-positive bacteria being most commonly implicated [12]. Additionally, *Mycobacterium tuberculosis* (TB) needs to be evaluated as part of the differential diagnosis of pericardial effusions, particularly when the clinical presentation is subacute. It should be highly suspected in patients who are from endemic regions or have underlying risk factors such as an HIV infection, have recently immigrated from high-risk regions, have been incarcerated, or have known prior TB exposure, regardless of geographic setting. Unlike bacterial PPE, tuberculous pericarditis produces a lymphocytic exudative effusion. TB is the most common cause of pericardial disease in high-burden settings, particularly in sub-Saharan Africa, where it accounts for the majority of cases in both HIV-infected and non-infected populations [13].

Furthermore, diagnosis relies on establishing tubercle bacilli in the pericardial fluid or, if tuberculosis is confirmed elsewhere, on a lymphocytic exudate and elevated adenosine deaminase (ADA) activity [14]. An ADA above 40 U/L has a pooled sensitivity of 88% and specificity of 83% for tuberculous pericarditis [15]. When confirmed, treatment usually follows standard multidrug anti-tuberculosis regimens, typically six months, with the first two months comprising isoniazid, rifampicin, pyrazinamide, and ethambutol, followed by four months of isoniazid and rifampicin. Adjunctive corticosteroids are not routinely recommended in pericardial TB [16].

Gram-negative pathogens, anaerobes, and fungal infections have increased in prevalence in recent years, however [17]. Polymicrobial infection, including combined bacterial and fungal involvement, is a particularly uncommon but clinically important variant of PPE, as it requires high suspicion for diagnosis and is fatal if missed. The organisms implicated are mainly attributable to the source of infection and the host's immune status, highlighting the importance of clinical background in guiding diagnostic testing and enabling early management. Conversely, the organisms isolated can point toward the possible route of infection and the underlying source. Gram-positive organisms usually spread hematogenously from a thoracic source, while gram-negative organisms, anaerobes, and fungal organisms are commonly seen in immunocompromised patients, from a gastrointestinal source or postoperatively. For instance, gram-negative bacilli are commonly isolated after gastrointestinal resection in patients with cancer due to the spread of colonic flora to the mediastinum in patients with poor immunity [18].

Predisposing conditions and procedural risk

Underlying infectious diseases were the main source of purulent pericarditis before antibiotics were widely used. This change was observed over time at John Hopkins Hospital, showing that before 1943, pneumonia was the underlying cause in more than half the cases through direct pulmonary extension. In contrast, cases in recent years occurred due to spread from the intrathoracic space, particularly after intrathoracic procedures [18]. Purulent pericarditis can occur due to an infection spreading to the pericardium through direct extension from the myocardium and intrathoracic space, whereby pathogens migrate from a neighboring infected structure, such as the mediastinum, lungs, or pleura, through adjacent tissue into the pericardial space without having to enter the bloodstream, or through a perforating injury to the chest wall, whether through trauma or percutaneous procedures. Another mode of transmission is hematogenous spread of the infection, which usually occurs in patients with chronic medical conditions that predispose them to immunocompromised states and can yield a variety of organisms.

The most implicated risk factors are an immunocompromised state, active malignancy, and invasive cardiothoracic procedures [7], including minimally invasive procedures such as percutaneous coronary intervention (PCI) [19]. Active malignancy is among the most common conditions predisposing patients to immunocompromise in the setting of PPE [20,21]. Still, it should also be considered in other settings, such as sickle cell disease, chronic corticosteroid therapy, and patients with multiple chronic medical and cardiovascular comorbidities [22-24]. Additional risk factors include contiguous spread from nearby structures, as seen in a previously described case of PPE in the context of a mycotic aortic pseudoaneurysm [25] and another case secondary to a mycotic coronary aneurysm following PCI with rotational atherectomy [26]. This has also been previously described in cases of infective mural endocarditis [27]. While less common, these alternative risk factors and associated conditions should be acknowledged, as the presentation may be indolent, difficult to culture and identify, and usually on a background of immune dysfunction. Consequently, empiric treatment covering multiple organisms should remain broad early on.

Clinical presentation and course

The presentation and course of PPE can be quite variable, although overall outcomes remain significantly worse than in cases of non-purulent pericardial effusion [5]. The early clinical presentation can resemble pneumonia or sepsis of unclear origin, due to the nonspecific symptoms. Across two retrospective reviews of purulent pericarditis cases, both reported fever as the most common finding, at 85% and 100%, respectively [28,29]. Dyspnea was also a highly sensitive finding, but typical pericarditis features, such as chest pain and an audible friction rub, were only present in about 37% of patients [28,29]. This underscores the nonspecific nature of the early presentation of PPE. As pericardial fluid accumulates rapidly, cardiac tamponade with resultant hemodynamic collapse may occur. Physical examination is typically normal when there is no hemodynamic instability. Overt findings of tamponade include hypotension, jugular venous distension, distant heart sounds, and pulsus paradoxus with bedside blood pressure measurement [30].

Left untreated, PPE is typically rapidly progressive and highly fatal, with obstructive cardiogenic shock and death occurring within hours to a few short days after diagnosis [20,31,32]. Progression to constrictive pericarditis, even after appropriate treatment, may occur in up to 30% of cases because of dense fibrin deposition and pericardial inflammation, further contributing to the morbidity associated with this condition [17,33]. Another important consideration is the possibility of pericardial fluid reaccumulation, which often requires surgical intervention, making serial imaging after successful drainage paramount in the management of PPE [34].

Diagnosis and diagnostic challenges

When to Suspect PPE

Given its rarity, variable presentation and progression, and need for invasive testing to establish a diagnosis, PPE poses significant diagnostic challenges. This makes timely recognition and early treatment difficult, contributing to the morbidity and mortality seen [35]. Delayed diagnosis remains a major contributor to morbidity and mortality with PPE, especially when early clinical findings overlap with several common diagnoses. For instance, chest pain and dyspnea commonly signify workup for acute coronary syndrome or pulmonary embolism rather than an inflammation of the pericardium. PPE should be suspected when the clinical presentation is severe and rapidly progressive, with pericardial effusion findings on imaging, especially in the context of recent thoracic procedures, malignancy, immunocompromised status, or a distant source of infection.

Imaging Findings

No single imaging modality can provide a definitive diagnosis of PPE; usually, a multimodality diagnostic workup will be required. Chest radiographs may show pleural effusion, cardiomegaly, or evidence of a pulmonary source of infection, yet findings are considered nonspecific and can be seen in other conditions. Also, if the effusion is small, the chest X-ray can appear completely normal, making it an unreliable imaging modality on its own for ruling in or out PPE. Moreover, given its bedside availability, echocardiography is the preferred initial imaging for assessment of PPE. The effusion may demonstrate heterogeneity with echogenic material, loculations, or fibrinous strands. It can also show features of a tamponade, right ventricular diastolic collapse, a plethoric inferior vena cava, and respiratory variation in mitral and tricuspid inflow velocities; these should be assessed in every patient [36]. Additionally, transesophageal echocardiography can provide a more detailed examination of posterior or loculated collections when transthoracic views are limited. Chest CT is considered superior to echocardiography for more anatomical detail. Pericardial effusion in PPE has a density similar to that of blood on CT imaging [36,37]; CT also aids surgical planning. Cardiac MRI provides good visualization of soft tissues but is less useful acutely, given the time required for the entire process; as such, it is better reserved for follow-up assessment or when CT findings are not clear [36].

Pericardial Fluid Analysis

If there is significant effusion, tamponade, or suspected PPE, urgent pericardiocentesis is needed as it is both diagnostic and therapeutic. The fluid is analyzed for the white cell count with differential, glucose, protein, lactate dehydrogenase (LDH), gram stain, bacterial culture, and fungal organisms in suspected cases. Blood cultures can also be useful in identifying the organisms from the primary source of infection, which is usually the same as the infected pericardial fluid. Pericardial fluid may be frankly purulent upon aspiration [38]. Light's criteria, often used to classify pleural effusions, have previously been shown to be beneficial for diagnosing PPE, with a diagnostic accuracy of 87% for an elevated fluid: serum lactate dehydrogenase ratio > 0.6, a low fluid-to-serum glucose ratio, and elevated pericardial fluid WBC count with neutrophilic predominance may further support the diagnosis and can help differentiate PPE from tuberculous and malignant pericarditis [39]. Although pericardial fluid analysis is useful for diagnosis, it is important to be cautious about relying solely on fluid results, as normal pericardial fluid can have elevated LDH, protein, and lymphocyte counts [40,41].

In cases where pericardiocentesis produces a milky and opaque appearance, chylopericardium should be suspected. It is commonly caused by injuries of the thoracic duct from surgery or trauma, and by malignancy, specifically lymphoma. An elevated triglyceride content, a cholesterol-to-triglyceride ratio below 1, and fat globules on Sudan staining further support the diagnosis [42]. Moreover, as this can be associated with malignancy, it is crucial to send cytology as part of the workup, followed by MRI or CT to identify the underlying cause, along with lymphangiography, which may further localize the site [43].

Diagnostic pitfalls and differential diagnosis

Due to the severe febrile illness of this condition, sepsis could be the predominant manifestation, and an erroneous diagnosis is often made. In a presentation of chest pain, fever, and dyspnea, clinicians are more likely to suspect common diagnoses such as pneumonia, acute coronary syndrome, pulmonary embolism, and viral pericarditis. Moreover, of the several etiologies of pericarditis, inflammatory and viral etiologies are more common in practice than bacterial, which accounts for less than 1% of cases [30]. Additionally, purulent pericardial effusion may be missed because the aspirated fluid may not be frankly purulent, even though the underlying source is bacterial, leading to an incorrect ruling out of a serious etiology. In these cases, with nonspecific presentations, diagnosis may be delayed, potentially precipitating tamponade and making effective drainage difficult, leading to recurrence. The primary issue is that, even after pericardiocentesis, there is no official diagnostic criterion for distinguishing between transudative and exudative etiologies. Light's criteria, used to analyze pleural fluid, have been clinically adapted to analyze pericardial fluid to help guide medical management of pericardial effusions [39]. The accuracy of applying this criterion is questionable, as pericardial fluid has different baseline characteristics than pleural fluid. Light's criteria classify pleural fluid as exudative when the fluid protein-to-serum ratio is above 0.5, the fluid LDH-to-serum ratio is >0.6, or the fluid LDH is two-thirds of the serum's upper limit. In comparison, normal pericardial fluid is rich in LDH, protein, and lymphocytes; thus, analyzing it according to Light's criteria would overestimate it as exudative and has little value in determining the underlying etiology [40]. This was confirmed by a study analyzing pericardial fluid characteristics in 30 patients without pericardial disease, providing a reference for the composition of normal pericardial fluid. They found that LDH was 2.4 times the serum level, protein was 0.6 times the serum level, and leukocyte count was 1,430/μL, predominantly lymphocytes [40].

Management

Initial Stabilization

In this rapidly progressive condition, patients must be assessed for hemodynamic instability and managed as an emergency. If the patient is hemodynamically unstable, immediate echography-guided drainage is needed with an indwelling catheter placed for continuous fluid drainage, along with hemodynamic support and involvement of cardiology, cardiothoracic surgery, and infectious diseases for further management. Source control is the mainstay; thus, drainage and intravenous antibiotics must not be delayed. The 2015 European Society of Cardiology (ESC) guidelines further emphasize aggressive management with empiric antibiotics and drainage, as death will ensue otherwise [30].

Drainage Strategies

Pericardiocentesis is initially required when a patient is hemodynamically unstable and can be sufficient to eradicate the infection if performed early; however, in the majority of PPE cases, the fluid can be fibrinous and loculated, reaccumulate, and result in pericardial empyema. According to the European Society of Cardiology, a surgically placed indwelling catheter can be used for a purulent effusion, with a class 1c recommendation. This is known as a subxiphoid pericardiocentesis, which involves irrigation with saline at intervals and, if necessary, antimicrobials. Fibrinolytic drugs, such as streptokinase, are also used locally when the fluid is thick and cannot be drained effectively with a catheter alone. Fibrinolytic agents can dissolve fibrin and are thought to be most effective when administered within the first week of presentation, before established fibrosis develops [7,44-46].

Antimicrobial Therapy

Culture-specific antimicrobials are another important cornerstone of treatment, although guidelines are insufficient on the duration of antibiotics. A general rule is to use broad-spectrum antibiotics initially to cover common causative organisms, such as *Staphylococcus aureus*, then narrow therapy based on culture and sensitivity results. Initial antibiotic coverage depends on the route of infection, the patient's immune status, and a history of recent procedures. Broader coverage would be reasonable in patients with malignancy, suspected spread from an intra-abdominal source, and recent thoracic or gastrointestinal procedures. Anti-fungals are not regularly required in all cases but are considered in certain scenarios, such as patients who are severely immunocompromised and have failed to improve with broad-spectrum antimicrobials. Antibiotics should be continued until the source of infection is completely eradicated, and the patient should be monitored for clinical improvement in conjunction with biochemical improvement. This is a complicated infection; antibiotics are usually required for at least a few weeks to ensure resolution [46,47].

Effusive-constrictive pericarditis (ECP)

Effusive-constrictive pericarditis (ECP) should be suspected when there remains a persistently elevated jugular venous pressure or equalization of diastolic filling pressures after pericardiocentesis. It is characterized by the failure of right atrial pressure to fall below 10 mmHg, or at least by 50%, after normalization of intrapericardial pressure [48]. In PPE, effusive-constrictive pericarditis (ECP) can persist despite drainage of the effusion, due to fibrin deposition and ongoing inflammation. This must be managed by continuing targeted antimicrobial therapy with close hemodynamic monitoring. Furthermore, pericardiectomy was required for persistent constriction in the original prospective series, while three patients had spontaneous resolution without the need for surgery [48].

Surgery and follow-up

**Figure 1:**
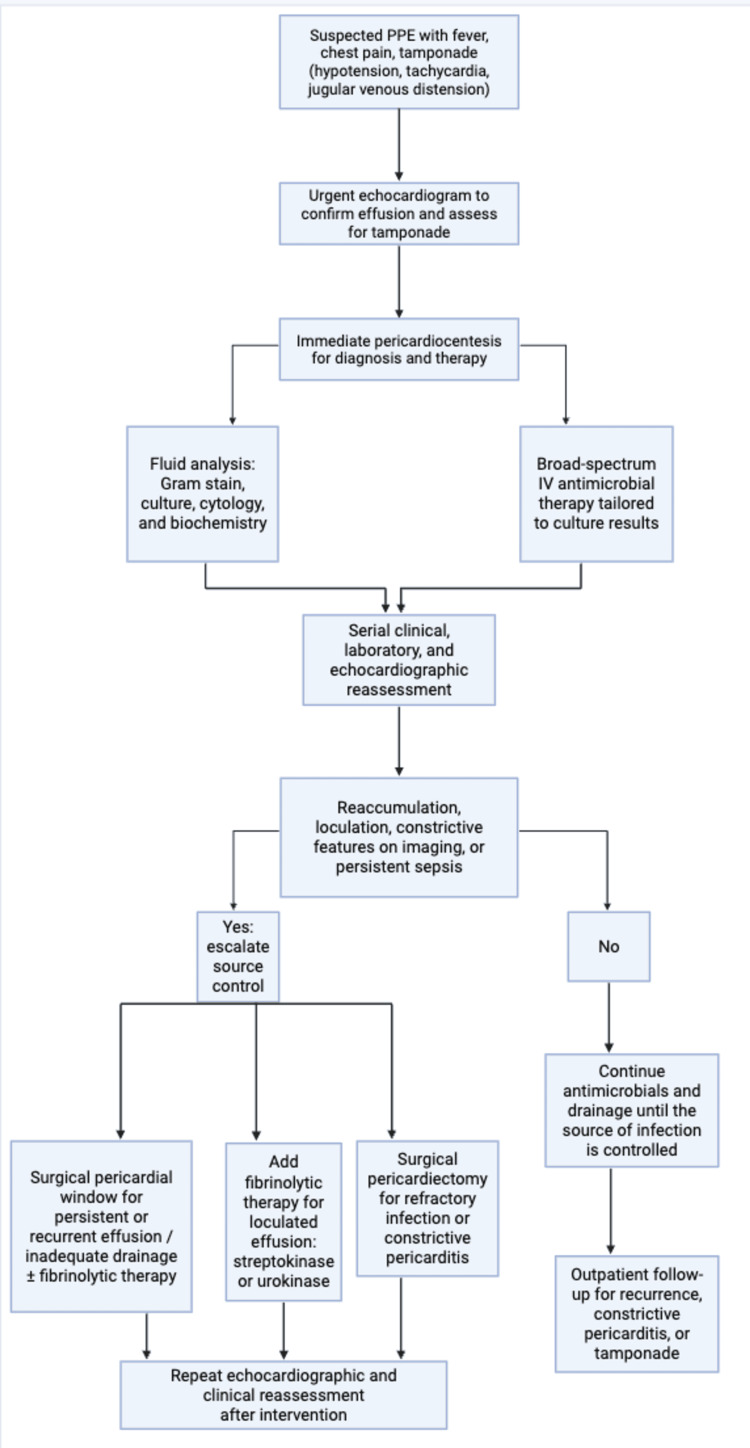
Proposed diagnostic and management approach to suspected purulent pericardial effusion PPE: purulent pericardial effusion, IV: intravenous The figure was created using the BioRender website.

Surgical methods are indicated if there is evidence of dense adhesions, recurrent tamponade, persistent infection, an encapsulated effusion, and progression to constrictive pericarditis. Fibrin formation is the reason effusion persists, which calls for the need for surgical intervention to prevent constrictive pericarditis from resulting. Thus, repeat echocardiographic imaging and monitoring for recurrence are vital despite initial hemodynamic improvement after drainage. Clinical deterioration and persistence of fever should prompt reevaluation for resistant organisms, reaccumulation of fluid, and escalation to surgical intervention. Early recognition of inadequate treatment is vital in such a critical case that has a fulminant course. Currently, the European Society of Cardiology recommends (class 1c) a pleuropericardial window for a purulent effusion that is heavily loculated and persists despite conservative medical measures [30]. Surgical drainage via a pericardial window may be considered, as PPE is likely to recur. This is created endoscopically or via thoracotomy to connect the pericardium to the left pleural space, allowing drainage. Additional intrapericardial thrombolysis can be considered to reduce dense loculations before surgical intervention. Although there is insufficient data to conclude the efficacy of thrombolytic therapy, a systematic review of 40 cases of purulent pericarditis treated with fibrinolytic therapy found that only two patients developed constrictive pericarditis [46]. Those two cases were treated with late fibrinolysis and required a pericardiectomy. It is a minimally invasive alternative to surgery, but a pericardiectomy is the main option when fibrinolysis fails. A pericardiectomy is a surgical option that seems to be more effective in completely eradicating the infection and thus preventing constrictive pericarditis. Despite this, pericardiectomy is usually reserved as a last resort because of its invasive nature and requires a thoracotomy [30,46]. Figure 1 presents the proposed diagnostic and management plan for a purulent pericardial effusion, including all possible treatment options.

Table 1 summarizes reports and case series on purulent pericardial effusion in adult patients from 2004 to 2025. Most of the patients presented with severe manifestations, including cardiac tamponade, sepsis, and shock, or continued to have purulent drainage from their chest. Furthermore, the presumed source of the purulent pericardial effusion could be identified in a few instances, such as respiratory infections, including pneumonia, mediastinitis, cavitary lung disease, or adjacent spread of an infectious process to the heart. The majority of cultures from patients with purulent pericardial effusions were positive for gram-positive bacteria, predominantly *Staphylococcus aureus* and *Streptococcus* species, which accounted for almost all gram-positive organisms. Polymicrobial infections were less common than monomicrobial infections; when they did occur, they were more complicated. Management of pericardial fluid was by pericardiocentesis, pericardial window, or partial pericardiectomy, combined with empirical antibiotic therapy targeting the pathogens identified on culture. It led to the successful management of this complication. The analysis points to the importance of early diagnosis of purulent pericardial effusion and adequate management to prevent complications such as constrictive pericarditis, mediastinitis, and prolonged hospitalizations.

**Table 1:** Published adult cases of purulent pericardial effusion and their clinical presentation, causative organisms, treatment, and outcomes The reviewed cases [7,28,44,49,50-54] highlight purulent pericardial effusion as a rare but severe condition, typically bacterial in origin, that usually presents with cardiac tamponade or sepsis and requires immediate pericardial drainage, along with prolonged antimicrobial treatment. COPD: chronic obstructive pulmonary disease, ICU: intensive care unit

Author/year	Study	Risk factor/medical history	Presumed source	Organism(s)	Clinical presentation	Management	Antimicrobial therapy	Outcome
Berrios et al. (2025) [49]	Case report	Diabetes mellitus	No source identified	Streptococcus pneumoniae	Initially presumed idiopathic acute pericarditis, later progressed to cardiac tamponade	Pericardiocentesis	Empiric vancomycin and piperacillin-tazobactam, later ceftriaxone	Developed constrictive pericarditis requiring partial pericardiectomy, with subsequent resolution
Pratama et al. (2022) [44]	Case report	Recent cardiac tamponade 3 weeks earlier	No source identified	Staphylococcus aureus	Recurrent pericarditis with cardiac tamponade	Pericardiocentesis with intrapericardial fibrinolytic therapy (1.5 million units)	Empiric ceftriaxone, followed by intravenous gentamicin for 14 days	No complications
Costa et al. (2021) [50]	Case report	COPD and previously treated pulmonary tuberculosis	Respiratory infection	*Streptococcus anginosus* and *Fusobacterium* species	Pericarditis	Pericardiocentesis	Empiric piperacillin-tazobactam and vancomycin, then penicillin G and clindamycin after culture results; later meropenem and vancomycin for 28 days after mediastinal abscess removal	Mediastinitis secondary to abscess formation
Kaye et al. (2019) [51]	Case report	No identified risk factors	No source identified	Staphylococcus aureus	Sepsis	Pericardial drainage	Empiric vancomycin and piperacillin-tazobactam, followed by cefazolin	No complications
Wada et al. (2014) [52]	Case report	No identified risk factors	Pneumonia	Gram-positive cocci	Pneumonia with associated purulent pericardial effusion	Pericardiocentesis followed by pericardial window on day 8	Ceftriaxone	Developed constrictive pericarditis 11 days after discharge
Petcu et al. (2013) [7]	Case report	Hodgkin disease on chemotherapy	No source identified	No growth	Epigastric pain radiating retrosternally	Pericardial drainage via left anterior thoracotomy with partial anterior pericardiectomy	Intravenous oxacillin, ceftriaxone, and gentamicin, with additional intrapericardial gentamicin	No complications
Parikh et al. (2009) [28]	Case report	Intravenous drug use, diabetes, and hepatitis C	Mycotic aneurysm of the aortic root	*Candida glabrata*, *Streptococcus agalactiae*, and *Propionibacterium acnes*	Cardiac tamponade	Pericardiocentesis followed by pericardial window	Intravenous fluconazole and penicillin for 7 weeks	No complications
Parikh et al. (2009) [28]	Case report	Prior splenectomy	Cavitary lung nodules	Methicillin-resistant *Staphylococcus aureus*	Cardiac tamponade	Pericardiocentesis followed by pericardial window	Intravenous vancomycin for 6 weeks	No complications
Leoncini et al. (2006) [53]	Case series/retrospective review	Previously healthy adults; one pregnant patient, one with prior serous pericarditis, and one with recent prostatitis	Two primary cases; the remainder were associated with pulmonary or mediastinal infection, or possible hematogenous spread after prostatitis	Beta-hemolytic *Streptococcus* with *Bacteroides fragilis* in one case; *Streptococcus* in two cases; *Streptococcus pneumoniae* in one case; *Pseudomonas aeruginosa* in one case	Delayed diagnosis in all cases, with tamponade and shock; dyspnea and pleural effusions were common, and one case had pyo-pneumo-pericardium	Thoracotomy with partial pericardiectomy in all patients; preoperative catheter drainage failed in two patients, and one required laparotomy	Antibiotics administered	All patients were discharged well; mean postoperative stay was 30.4 days, with follow-up ranging from 5 months to 12 years and no constrictive pericarditis reported
Tomkowski et al. (2004) [54]	Case series	Paratonsillar abscess in one patient; rheumatoid arthritis on prolonged corticosteroids in one patient; pneumonia with mediastinitis in one patient	Contiguous spread from neck infection in one patient; immunosuppression-related infection in one patient; direct spread from pneumonia or mediastinitis in one patient	*Streptococcus* species in one patient; *Streptococcus intermedius* in one patient; no growth in one patient	ICU admission with purulent pericarditis, persistent purulent drainage, and loculations or fibrin on echocardiography	Pericardiotomy with intrapericardial streptokinase	Antibiotics administered	No complications

Critical appraisal of published cases

The cases presented in Table 1 span 2004-2025 and reveal important patterns regarding certain outcomes and management. The two cases from Parikh et al. [28] show that fungal and polymicrobial infections can be missed, leading to potentially fatal consequences without a broad initial workup. Although the available data are insufficient to draw firm conclusions about specific organisms' outcomes, the cases complicated by constrictive pericarditis and mediastinitis were mostly associated with underlying immune dysfunction, delayed drainage, or inadequate source control, which suggests that these factors contribute to worse outcomes, yet this cannot be confidently assumed from case-level data by itself. Among the cases reviewed, several factors associated with a worse prognosis included presenting with septic shock and cardiac tamponade, which are consistent with previously identified poor prognostic factors [47]. Furthermore, prompt pericardiocentesis with catheter drainage seems to be the most beneficial initial approach, while the use of intrapericardial fibrinolytics, as used in the study by Tomkowski et al. (2004) [54] and Pratama et al. (2022) [44], represented a promising addition when fluid is viscous or loculated. In some cases, surgical escalation was required even after percutaneous drainage, which shows the importance of early surgical review. The main limitation across all entries is the small series or case report design, which carries risks of incomplete follow-up, selection bias, and heterogeneous management, thereby impeding reliable comparisons.

The figure illustrates an approach for the evaluation and treatment of a suspected PPE. The algorithm begins with a patient presenting with clinical suspicion symptoms, including fever, chest pain, and features of tamponade (hypotension, tachycardia, and jugular venous distension). This prompts an urgent echocardiogram to confirm the presence of an effusion and investigate for any underlying tamponade physiology. After which, when PPE is suspected, an immediate pericardiocentesis is performed as both a diagnostic and treatment option, along with starting broad-spectrum intravenous antibiotics. Pericardial fluid and blood cultures are sent to guide specific therapy. After the initial drainage, the treatment course branches based on clinical response, which is assessed through serial laboratory, clinical, and echocardiographic reassessment. In patients without reaccumulation, loculation, sepsis, or constrictive features, drainage and antimicrobials are continued until the infection is controlled. Follow-up in the outpatient setting is required to look for any signs of recurrence, tamponade, or constrictive pericarditis. However, if patients fail to respond to initial treatment measures, source control is escalated through one of three options, depending on the clinical scenario. These include a surgical pericardial window for recurrent or persistent effusion without proper drainage, intrapericardial fibrinolytic therapy with streptokinase or urokinase for loculated effusions, or surgical pericardiectomy for refractory infection or if it progresses to constrictive pericarditis. The three pathways lead to repeating clinical and echocardiographic reassessment after the interventions to ensure no recurrence and to monitor patients for any signs of late constriction closely.

Current gaps in the literature

Published evidence on purulent pericardial effusion remains limited, largely because most available literature consists of case reports and small case series, making it difficult to establish valid clinical practice recommendations. For this reason, there are still no guidelines on the optimal duration of antimicrobial therapy, clear indications for early surgical intervention, and established risk factors for predicting recurrence or progression to constrictive pericarditis. There are several non-surgical and surgical management options, but there is insufficient evidence on the appropriate intervention for managing cases. Furthermore, many papers lack long-term follow-up, leaving readers with little information on the optimal long-term management strategy.

Thus, creating an international reporting registry and conducting larger pooled analyses on the condition are required to improve diagnostic accuracy and therapeutic strategies.

Limitations

This review has various limitations. As this is a narrative review, it cannot be guaranteed that all relevant literature can be captured. Most of the evidence base stems almost entirely from small series and case reports, which are prone to publication and selection bias. Moreover, there is variability across cases in patient backgrounds, causative organisms, treatment approaches, and follow-up protocols, making statistical synthesis difficult; most conclusions present descriptive observations rather than established findings. Additionally, long-term outcomes are not fully reported in much of the literature, and variation in PPE microbiology limits the generalizability of the data. All in all, these limitations are common in rare diseases, which reinforces the need for standardized reporting and an international registry.

## Conclusions

PPE is a rare but serious medical condition that presents as a rapidly progressive cardiac emergency with high rates of mortality and significant associated morbidity both in the short term and the long term. A high index of suspicion should be employed in any patient presenting with a rapidly progressive febrile pericarditis condition with associated pericardial effusion, particularly if imaging suggests a heterogeneous, fibrinous, or complex effusion. Early pericardiocentesis and antimicrobial therapy are prudent, with surgical intervention not uncommonly needed. Close follow-up is crucial for early identification and management of constrictive pericarditis. Standardized reporting and larger pooled analyses are required to define a standardized diagnostic approach, appropriate antimicrobial duration, and follow-up strategies.
